# GAIA: An easy-to-use web-based application for interaction analysis of case-control data

**DOI:** 10.1186/1471-2350-7-34

**Published:** 2006-04-05

**Authors:** Stuart Macgregor, Imtiaz A Khan

**Affiliations:** 1 Genetic Epidemiology, Queensland Institute of Medical Research, Brisbane, Australia; 2 Biostatistics and Bioinformatics Unit, Cardiff University, Cardiff, UK

## Abstract

**Background:**

The advent of cheap, large scale genotyping has led to widespread adoption of genetic association mapping as the tool of choice in the search for loci underlying susceptibility to common complex disease. Whilst simple single locus analysis is relatively trivial to conduct, this is not true of more complex analysis such as those involving interactions between loci. The importance of testing for interactions between loci in association analysis has been highlighted in a number of recent high profile publications.

**Results:**

Genetic Association Interaction Analysis (GAIA) is a web-based application for testing for statistical interactions between loci. It is based upon the widely used case-control study design for genetic association analysis and is designed so that non-specialists may routinely apply tests for interaction. GAIA allows simple testing of both additive and additive plus dominance interaction models and includes permutation testing to appropriately correct for multiple testing. The application will find use both in candidate gene based studies and in genome-wide association studies. For large scale studies GAIA includes a screening approach which prioritizes loci (based on the significance of main effects at one or both loci) for further interaction analysis.

**Conclusion:**

GAIA is available at

## Background

Genetic association mapping is one of the primary tools used to identify loci involved in common complex disease. Such analyses are typically implemented by testing for a difference between allele frequencies at a locus in a population sample of cases and controls. However, such an approach only considers one locus at a time. Most common diseases will be genetically complex, with multiple loci contributing to disease susceptibility. Epistasis is the phenomenon where the phenotypic effect of one locus changes as a result of the genotype at one or more other loci. The importance of epistasis has been strongly emphasised recently [1-3], with the poor replication rate of human genetic association studies cited as being partly attributable to the lack of consideration given to epistatic effects [4,5]. Another recent paper [6] has suggested the power of large scale studies may be substantially improved by considering interactions among loci.

Appropriate analysis of population data may be invaluable in identifying loci that exhibit significant interaction (in the statistical sense). Although analyses which consider interaction terms can be implemented in packages such as R [7] or STATA (for scripts and further details see [8]), such analyses are difficult for non-specialists to implement and cannot be readily applied to large numbers of genetic markers. Since large volumes of population data are now being generated in many molecular genetics laboratories there is an urgent need for applications which can streamline the data analysis stage of genetic association mapping projects. GAIA, a freely available, easy-to-use web application, allows non-specialist users to routinely test for interactions.

## Methods

### Regression model

GAIA uses perl CGI scripts to code the data, with the R package [7] used for the necessary statistical routines. The application uses a regression model which allows the user to test for pairwise locus-locus interactions between genes. For the case-control data sets typically employed, this utilizes the logistic regression model

log(p1−p)=μ+a1x1+d1z1+a2x2+d2z2+iaax1x2+iadx1z2+idax2z1+iddz1z2
 MathType@MTEF@5@5@+=feaafiart1ev1aaatCvAUfKttLearuWrP9MDH5MBPbIqV92AaeXatLxBI9gBaebbnrfifHhDYfgasaacH8akY=wiFfYdH8Gipec8Eeeu0xXdbba9frFj0=OqFfea0dXdd9vqai=hGuQ8kuc9pgc9s8qqaq=dirpe0xb9q8qiLsFr0=vr0=vr0dc8meaabaqaciaacaGaaeqabaqabeGadaaakqGabeqaeqXbbaacbiGae8hBaWMae83Ba8Mae83zaC2aaeWaaeaadaWcaaqaaiabdchaWbqaaiabigdaXiabgkHiTiabdchaWbaaaiaawIcacaGLPaaacqGH9aqpiiGacqGF8oqBcqGHRaWkcqWGHbqydaWgaaWcbaGaeGymaedabeaakiabdIha4naaBaaaleaacqaIXaqmaeqaaOGaey4kaSIaemizaq2aaSbaaSqaaiabigdaXaqabaGccqWG6bGEdaWgaaWcbaGaeGymaedabeaakiabgUcaRiabdggaHnaaBaaaleaacqaIYaGmaeqaaOGaemiEaG3aaSbaaSqaaiabikdaYaqabaGccqGHRaWkcqWGKbazdaWgaaWcbaGaeGOmaidabeaakiabdQha6naaBaaaleaacqaIYaGmaeqaaaGcbaGaaCzcaiabgUcaRiabdMgaPnaaBaaaleaacqWGHbqycqWGHbqyaeqaaOGaemiEaG3aaSbaaSqaaiabigdaXaqabaGccqWG4baEdaWgaaWcbaGaeGOmaidabeaakiabgUcaRiabdMgaPnaaBaaaleaacqWGHbqycqWGKbazaeqaaOGaemiEaG3aaSbaaSqaaiabigdaXaqabaGccqWG6bGEdaWgaaWcbaGaeGOmaidabeaakiabgUcaRiabdMgaPnaaBaaaleaacqWGKbazcqWGHbqyaeqaaOGaemiEaG3aaSbaaSqaaiabikdaYaqabaGccqWG6bGEdaWgaaWcbaGaeGymaedabeaakiabgUcaRiabdMgaPnaaBaaaleaacqWGKbazcqWGKbazaeqaaOGaemOEaO3aaSbaaSqaaiabigdaXaqabaGccqWG6bGEdaWgaaWcbaGaeGOmaidabeaaaaaa@7BC5@

where p is the probability of each individual being a case, *x*_*i *_and *z*_*i *_are dummy variables with *x*_*i *_= 1, *z*_*i *_= -0.5 for one homozygote genotype, *x*_*i *_= 0, *z*_*i *_= 0.5 for the heterozygote genotypes and *x*_*i *_= -1, *z*_*i *_= -0.5 for the other homozygote [9]. We assume a diallelic locus such as a single nucleotide polymorphism (SNP). *μ *corresponds to the mean effect. The terms *a*_1_, *d*_1_, *a*_2_, *d*_2 _represent the parameters corresponding to the additive and dominance effects at the two SNPs (i.e. the main effects). Similarly, *i*_*aa*_, *i*_*ad*_, *i*_*da*_, *i*_*dd *_represent the epistatic interaction effects.

### Implemented tests

GAIA allows an "additive plus dominance" 4 degree of freedom (df) test of interaction in which a model with the terms *i*_*aa*_, *i*_*ad*_, *i*_*da*_, *i*_*dd *_is compared with one without them; in GAIA this is referred to as the "interaction only" p-value. The "interaction only" p-value tests the significance of the interaction model terms over and above any main effects. Also implemented is an 8 df test of overall significance (model with all terms compared with model with only *μ *fitted); in GAIA this is referred to as the "overall" p-value. The "overall" p-value tests the significance of all terms in the model (i.e. the joint significance of both main effects and interaction effects). By dropping dominance terms GAIA can perform an "additive only" 1 df interaction test (i.e. a model with *a*_1_*x*_1_, *a*_2_*x*_2 _and *i*_*aa *_versus a model with *a*_1_*x*_1 _and *a*_2_*x*_2_) and a 3 df overall significance test (i.e. a model with *a*_1_*x*_1_, *a*_2_*x*_2 _and *i*_*aa *_versus a model with only *μ*). The significance of the relevant model terms can be evaluated by comparing twice the log-likelihood difference between models with a *χ*^2 ^distribution. Alternatively, permutation based tests can be applied (see below). The parsimonious models with only additive effects will be powerful when dominance effects are moderate or small. For markers with small minor allele frequencies it may not be possible to fit all the interaction terms with the additive plus dominance model; this results because some of the relevant locus-locus genotype are not present in the data. In general, with *n *and *m *model terms for each marker, the interaction test will have *n *× *m *df. This means that interaction tests involving multiallelic markers and/or haplotypes will have large numbers of degrees of freedom. Tests based on large df are unlikely to powerful for general screening of genes and hence are not implemented in the web application. Multiallelic markers can of course be downcoded to two alleles for use in the application. GAIA is intended for use with genes that are in linkage equilibrium (for example genes far apart on the same chromosome or on different chromosomes). For nearby gene pairs the application still gives valid results but this sort of data is probably better dealt with by constructing haplotype based tests of association.

### Input format

GAIA has a flexible input format allowing either i) the data for both genes to be coded in a single file, or ii) separate data files for each gene; files are automatically merged based on matching values in the first field. The input file is required to be in "Linkage" format, which is essentially the de facto standard for coding genetic data [10]. The user then specifies the marker(s) of interest and the analysis options required. In addition to additive only and additive plus dominance interaction models the program can output standard allelic (i.e. additive only) and genotypic (i.e. additive plus dominance) tests for each SNP singly.

### Permutation testing

Although Bonferroni corrections can be readily applied for independent statistical tests, markers within the same gene are likely to have correlated allele frequencies. We hence apply a permutation procedure which appropriately takes into account this non-independence [11]. The two different possible significance tests require different permutation tests. The test for the significance of the interaction terms (over and above the main effects) is performed by permuting the interaction model terms, with the main effects and disease status remaining as in the original data set. The test for overall significance is performed by permuting the status variable and keeping the other model terms fixed. The possible permutation tests are discussed further in a paper by Carlborg and Andersson [12]. The relevant test statistics are recalculated a large number of times, with the appropriate permutation procedure applied each time. By sorting the resultant p-values we can calculate an empirical p-value. The permutation procedure can also be used to correct for the non-independence of the two possible interaction tests.

In the simple case where the permutation test is conducted on a single marker, the permutation p-values should be very similar to the (asymptotic) p-values from a *χ*^2 ^distribution on the appropriate degrees of freedom. We therefore validated the "interaction only" test by comparing the asymptotic p-value (from a χ12
 MathType@MTEF@5@5@+=feaafiart1ev1aaatCvAUfKttLearuWrP9MDH5MBPbIqV92AaeXatLxBI9gBaebbnrfifHhDYfgasaacH8akY=wiFfYdH8Gipec8Eeeu0xXdbba9frFj0=OqFfea0dXdd9vqai=hGuQ8kuc9pgc9s8qqaq=dirpe0xb9q8qiLsFr0=vr0=vr0dc8meaabaqaciaacaGaaeqabaqabeGadaaakeaaiiGacqWFhpWydaqhaaWcbaGaeGymaedabaGaeGOmaidaaaaa@3079@ distribution)with the permutation p-value calculated for the "additiveonly" 1 df "interaction only" test (i.e. a model with *a*_1_*x*_1_, *a*_2_*x*_2 _and *i*_*aa *_versus a model with *a*_1_*x*_1 _and *a*_2_*x*_2_). The test was applied to a set of 135 cases and 289 controls typed for a single SNP. The asymptotic p-value for the interaction test was 0.01169. With 200000 permutations the permutation based p-value was 0.01171 (approximate 95% confidence interval for stochastic error in this estimate (0.01122,0.01219)). This indicates that permuting the interaction terms (i.e *i*_*aa *_in the additive case) and recalculating yields an appropriate p-value. In practice, such a large number of permutations would not typically be necessary. In the more complicated case where multiple markers are used, we would hence expect the permutation procedure to provide p-values that appropriately account for the multiple tests done.

## Results

### Candidate gene example

We used GAIA to analyse data from a case control study of the candidate gene GENEX in schizophrenia (real gene name suppressed). We wanted to test for interactions between GENEX and the gene GENEXInteractingProtein (which are on different chromosomes). There was a clear biological motivation for testing for interaction between SNPs in these two genes. We used GAIA to test 20 SNPs in GENEX for interactions with a SNP in GENEXInteractingProtein. 673 cases and 716 controls were available. The additive "interaction only" test was utilised. Applying GAIA to the available case control data yielded evidence for an interaction, with the p-value of 0.00033 for the significance of the interaction (over and above the main effects) for a marker in GENEX. A few of the 20 SNPs in GENEX were in strong linkage disequilibrium so applying a Bonferroni correction for the multiple markers tested would be overly conservative (Bonferroni corrected p-value was 0.0066). Applying the web applications permutation correction for multiple testing yielded a p-value of 0.0058. In a test run on this data set, 10000 permutations took ≃ 10 hours with GAIA. It is interesting to note that, when we examined GENEX and GENEX-InteractingProtein, we did not find significant main effects for either of the two SNPs that were found to interact (although we did find significant main effects for some of the other SNPs in GENEX).

### Large scale analysis

With approximately 30000 human genes and hence potentially (300002)
 MathType@MTEF@5@5@+=feaafiart1ev1aaatCvAUfKttLearuWrP9MDH5MBPbIqV92AaeXatLxBI9gBaebbnrfifHhDYfgasaacH8akY=wiFfYdH8Gipec8Eeeu0xXdbba9frFj0=OqFfea0dXdd9vqai=hGuQ8kuc9pgc9s8qqaq=dirpe0xb9q8qiLsFr0=vr0=vr0dc8meaabaqaciaacaGaaeqabaqabeGadaaakeaadaqadaqaauaabeqaceaaaeaacqaIZaWmcqaIWaamcqaIWaamcqaIWaamcqaIWaamaeaacqaIYaGmaaaacaGLOaGaayzkaaaaaa@33E0@ ≃ 45 million pairwise interactions, a large number of epistatic tests can be performed. In practice, the total number of tests done will be even greater than this because of the need to type multiple SNPs per gene. To reduce the multiple testing burden we suggest initially testing for interactions between candidate genes, as in the schizophrenia example above. We also recommend that, in the first instance, only pairs of SNPs with some evidence for main effects (at each SNP separately) are tested for interaction. Whilst it is possible that SNPs with smaller marginal effects (i.e. the effect of the SNP on its own) are important in higher order interaction terms, it makes sense to first test SNPs with significant main effects (see also screening approach below). Further discussion of interaction models with no main effects is given in [6,13,14].

In a wider, chromosome- or genome-wide context, there may also be value in applying interaction analysis, with the improved power outweighing the cost of the multiple-testing correction [6]. For large scale data GAIA can implement a screening approach. Loci are screened on a SNP by SNP basis with SNPs reaching a nominal level of significance (p < 0.05 for the additive single marker test) followed through to a secondary stage. The user can then apply one of the following approaches

1. test for interactions between the nominally significant SNPs

2. test for interactions between the nominally significant SNPs and all of the SNPs in the original data set

To perform this procedure in GAIA, the web application is first used to generate a file containing the nominally significant SNPs. This file is then either i) reloaded into GAIA in both input boxes (first approach), or ii) reloaded into one input box with the original data file loaded into the other input box (second approach). GAIA is then run as usual on the relevant subset of SNPs. Although both the "interaction only" and the "overall" test can be applied here, recent research suggests that utilising the "overall" test may be particularly fruitful here (see also discussion section). More detailed instructions on performing the screening procedure are included on the GAIA web page.

Although the first screening approach is computationally less intensive and requires fewer tests, we would recommend the second of the two approaches. This is because the increase in the number of tests is modest and it is rather restrictive to only test pairs where both have main effects. In many realistic scenarios where epistatic effects are important, the main effect of at least one of the interacting loci would not be significant [6] and hence should ideally not be discarded in the screening stage.

To test the feasibility of applying GAIA to a large number of markers we applied the screening approach to a set of 600 SNPs typed in 135 cases and 289 controls across chromosome 10. Testing for all possible pairwise combinations would involve 600 × 599/2 = 179700 interaction tests. Whilst not impossible, this number of tests on these data would take ≃ 15 hours computing time with GAIA. Applying the first and second screening approaches above reduced the number of interaction tests to 29×282
 MathType@MTEF@5@5@+=feaafiart1ev1aaatCvAUfKttLearuWrP9MDH5MBPbIqV92AaeXatLxBI9gBaebbnrfifHhDYfgasaacH8akY=wiFfYdH8Gipec8Eeeu0xXdbba9frFj0=OqFfea0dXdd9vqai=hGuQ8kuc9pgc9s8qqaq=dirpe0xb9q8qiLsFr0=vr0=vr0dc8meaabaqaciaacaGaaeqabaqabeGadaaakeaadaWcaaqaaiabikdaYiabiMda5iabgEna0kabikdaYiabiIda4aqaaiabikdaYaaaaaa@33A7@ = 406 and 600 × 29 - (29×302)
 MathType@MTEF@5@5@+=feaafiart1ev1aaatCvAUfKttLearuWrP9MDH5MBPbIqV92AaeXatLxBI9gBaebbnrfifHhDYfgasaacH8akY=wiFfYdH8Gipec8Eeeu0xXdbba9frFj0=OqFfea0dXdd9vqai=hGuQ8kuc9pgc9s8qqaq=dirpe0xb9q8qiLsFr0=vr0=vr0dc8meaabaqaciaacaGaaeqabaqabeGadaaakeaadaqadaqaamaalaaabaGaeGOmaiJaeGyoaKJaey41aqRaeG4mamJaeGimaadabaGaeGOmaidaaaGaayjkaiaawMcaaaaa@3522@ = 16965, respectively (29 of the 600 SNPs were significant at the 5% level). 16965 interaction tests (additive terms only model) took 80 mins to run, indicating that the screening approach allows large numbers of markers to be readily tested with GAIA.

With large numbers of markers the applications capacity for permutation analysis is limited. However, since a relatively small proportion of such loci will be correlated, the Bonferroni correction will not be overly conservative when applied to large numbers of markers (cf. situation with a small number of markers within a candidiate gene where LD may be strong and hence the Bonferroni correction rather conservative).

## Discussion

GAIA allows researchers to apply two different tests. One test, the "interaction only" test, considers the significance of the interaction terms on their own (over and above main effects). The other test, the "overall" test, considers the overall significance of both the main (or marginal) effects and the interaction effects together (i.e. a model with the terms *a*_1_, *d*_1_, *a*_2_, *d*_2_, *i*_*aa*_, *i*_*ad*_, *i*_*da*_, *i*_*dd *_compared with a model without them). The tests will be useful in different situations. The "interaction only" test will be most useful in candidate gene studies; for example in the schizophrenia data described here, there was evidence for statistical interaction between two biologically related genes. The "overall" test will be useful as a replacement for association testing of large numbers of loci singly. The "overall" test was discussed in this context by Marchini et al [6]; they show that models with interaction terms can be more powerful than simpler models which ignore interaction. Power improvements were shown both for a brute force approach which tested all possible interactions and an approach which screened loci for nominal significance [6]. In many realistic scenarios they show that the improved power outweighs the cost of the multiple-testing correction. Essentially, the increase in significance when fitting the "correct" model scales better with sample size than magnitude of multiple testing correction [3]. Models similar to those described by Marchini et al were considered recently by Millstein et al [15]. Millstein et al apply a slightly different set of sequential tests. Tests are done by selectively conditioning on previous results from single locus tests [15]. Another approach which addressed some of the same issues (but not in a human genetics context) is Carlborg and Andersson [12]. In GAIA the implemented approach for sequential testing involves applying the screening approach described in the previous section. Consider the 600 SNP example outlined earlier. With 600 SNPs, one would expect to find approximately 30 SNPs that were significant on their own (at a nominal 5% significance level and assuming only a small proportion of loci actually influence disease risk). A useful screen (similar to that described in strategy III from Marchini et al) with the "overall" test would therefore be to compute the 600 × 30 - (30×312)
 MathType@MTEF@5@5@+=feaafiart1ev1aaatCvAUfKttLearuWrP9MDH5MBPbIqV92AaeXatLxBI9gBaebbnrfifHhDYfgasaacH8akY=wiFfYdH8Gipec8Eeeu0xXdbba9frFj0=OqFfea0dXdd9vqai=hGuQ8kuc9pgc9s8qqaq=dirpe0xb9q8qiLsFr0=vr0=vr0dc8meaabaqaciaacaGaaeqabaqabeGadaaakeaadaqadaqaamaalaaabaGaeG4mamJaeGimaaJaey41aqRaeG4mamJaeGymaedabaGaeGOmaidaaaGaayjkaiaawMcaaaaa@3514@ = 17535 possible overall tests (assuming we test the ≃ 30 SNPs against all 600). To maintain an appropriate type I error one needs to correct for multiple tests done. In this example if we take the best p-value from any test done, we would (Bonferroni) correct for 17535+600 = 18135 tests. A detailed comparison of the different approaches described for large scale association analysis with interaction [6,12,15] would be an interesting area for further study.

Logistic regression based interaction has been utilised by a various authors [6,9,15]. Although this method can be applied using standard statistical packages, GAIA facilitates simple application of the method with the added advantage of permutation analysis and simple screening for inclusion of SNPs in the interaction test. A non-parametric alternative to parametric analyses such as logistic regression is Multifactor Dimensionality Reduction (MDR) [16]. The MDR approach avoids specifying a particular model for the interactions and instead bases its inferences on sets of "high" and "low" risk multilocus genotypes. This approach can be powerful for certain models of interaction with little or no main effects. However, for many realistic models of interaction, the MDR approach has been shown to be less powerful than approaches based on logistic regression [15].

GAIA does not currently accommodate family-based association design data. Tests analogous to the family based Transmission Distortion Test (TDT, [17] and refinements) can be conducted through the use of conditional logistic regression [18] and this accommodates the linear modeling of interactions. However, such methods are most powerful when there are informative transmissions from heterozygote parents and the use of highly polymorphic markers (with high heterozygosity) undesirably leads to large numbers of degrees of freedom in the tests for interactions described above. This, combined with the larger sample sizes usually available, means that case-control design is likely to be most suitable for interaction analysis. It is important to differentiate between biological epistasis (e.g. where two or more genes are involved in the same biological pathway and are jointly responsible for the end phenotype) and statistical epistasis (i.e. the deviation of the terms *i*_*aa*_, *i*_*ad*_, *i*_*da*_, *i*_*dd *_from zero in the linear model stated above). Biological epistasis occurs at the individual level whereas statistical epistasis necessarily is based upon populations. There is no direct relationship between these two definitions of epistasis and the existence of a number of possible parameterizations of the penetrances (parameters that define the genotype-phenotype relationship for binary traits) mean that the significance of the interaction terms maybe scale dependent [9]. In GAIA we utilise the log odds of the penetrance; this function is widely used in epidemiological studies and yields results comparable to those obtained from standard contingency tables when applied to single SNPs. For further discussion of the biological/statistical epistasis issue see [9,14].

## Conclusion

GAIA allows non-specialists access to interaction analysis of genetic association data. In our lab the application has allowed such users to routinely screen the candidate genes they are currently interested in against a set of established loci for a variety of genetically complex psychiatric diseases. By combining appropriate biological information on the genes underlying the detected statistical interactions, GAIA users should be able to better understand the aetiology of the disease under study. GAIA also allows interaction analysis to be applied on a larger scale. A practical screening facility which discards loci not showing main effects at either locus is provided in GAIA to make large scale analysis tractable.

## Availability and requirements

GAIA is accessible via  and is freely available for use by academics and non-academics. The source code for GAIA (perl and R) is available from the above URL. GAIA currently runs on 2 separate Intel based PCs (both accessible via the above URL).

## Competing interests

The author(s) declare that they have no competing interests.

## Authors' contributions

SM and IAK planned the study. SM and IAK wrote and tested the software. SM wrote the paper. All authors read and approved the final manuscript.

## Pre-publication history

The pre-publication history for this paper can be accessed here:


